# ‘The unexpected journey’: a qualitative interview study exploring patient and health professionals experiences of participating in the knee arthroplasty versus joint distraction study (KARDS)

**DOI:** 10.1136/bmjopen-2023-083069

**Published:** 2024-07-11

**Authors:** Susanne Arnold, Hemant Pandit, Julie Croft, Deborah D Stocken, David R Ellard, KARDS Trial Team, Hemant Pandit, Andrew Metcalfe, Ruben Mujica-Mota, Thomas Hamilton, Bernard van Duren, Hemant Sharma, David R Ellard, David Murray, Deborah Stocken, Dennis McGonagle, Hamish Simpson, Roberta Longo, Julie Croft, Rachel Kelly, Isabelle Smith, Ruchi Higham, Howard Collier, Susanne Arnold

**Affiliations:** 1 Warwick Clinical Trials Unit, Warwick Medical School, University of Warwick, Coventry, UK; 2 Chapel Allerton Hospital, Leeds, UK; 3 Leeds Institute of Rheumatic and Musculoskeletal Medicine, University of Leeds, Leeds, UK; 4 Leeds Institute for Clinical Trials Research, University of Leeds, Leeds, UK; 5 University Hospitals Coventry and Warwickshire NHS Trust, Coventry, UK

**Keywords:** QUALITATIVE RESEARCH, Knee, Clinical Trial, Musculoskeletal disorders

## Abstract

**ABSTRACT:**

**Objective:**

The aim of the knee arthroplasty versus joint distraction (KARDS) randomised trial was to investigate whether knee joint distraction (KJD) is non-inferior to knee arthroplasty, also known as knee replacement (KR). Here we report the findings from qualitative interviews that were part of the planned KARDS process evaluation.

**Design and methods:**

Semi-structured qualitative interviews with staff and participants in secondary care. Data were analysed using thematic content analysis.

**Findings:**

We were unable to complete the full-planned KARDS process evaluation as recruitment to the trial was closed early but key common themes emerged.

Eleven members of staff were interviewed from two KARDS sites (eight initial interviews just after site opening and three follow-up interviews at 12 months). Eleven KARDS participants (six KR and five KJD) were interviewed. One overarching theme emerged: ‘An unexpected journey’. This incorporated subthemes including ‘an important research question’, ‘a roller coaster ride’, ‘lessons learnt’, ‘managing expectations’ and ‘a slow recovery’. These encapsulate experiences of both staff and participants.

**Conclusion:**

The information that we were able to collect highlights that providing adequate and comprehensive information about all aspects of treatment including estimated timelines of recovery are essential in clinical trials of novel interventions. Incorporating a comprehensive rehabilitation package following KJD was a key learning. Process evaluations in these complex trials are essential to determine issues as early as possible so appropriate changes can be made to ensure participants have a smooth journey through the trial experience.

**Trial registration number:**

ISRCTN14879004.

**Strengths and limitations of this study**

- As the trial was closed early, we were unable to complete the planned full process evaluation of knee arthroplasty versus joint distraction.
- All of our interview data was from staff at two research sites and participants recruited from one research site.
- Apart from adapting the analysis, the qualitative work undertaken adhered to the original study design to meet the aims of the planned process evaluation.

## Introduction

 Knee osteoarthritis (OA) is a common musculoskeletal condition which causes pain, functional disability and significantly impacts on quality of life (QoL).^1^ Individuals with severe knee OA are typically offered knee replacement (KR) to relieve pain and improve movement and mobility. This treatment is both clinically and cost-effective,^2^ but the life span of artificial joints is limited, particularly in young and active patients. If a KR fails, revision surgery is a possibility but can be complicated, expensive and is associated with higher morbidity, mortality and poorer outcomes.^3 4^

The number of younger patients (≤55 years old) needing KR is increasing.^4^ Subsequently, the likelihood of needing revision surgery is higher in this population. Therefore, a treatment which can postpone time to first KR, without impacting on QoL or the opportunity to have a KR at a later date is very much needed.^5 6^

Knee joint distraction (KJD) has been proposed as an alternative to KR for this younger population. Further detail about the two surgical interventions is available in the knee arthroplasty versus joint distraction (KARDS) protocol paper,^7^ but in brief, the aim of KJD is to induce tissue structure modifications, providing cartilage repair and normalisation of subchondral bone abnormalities.^8^ It is a surgical procedure which involves placing an external fixator across a synovial joint to progressively pull apart the two osteochondral ends of the joint over a period of 3 days, through a total distance of 5 mm, which is maintained for a 6 week period.^9^ Initial studies from the Netherlands suggest that KJD is a safe and potentially effective treatment,^10^,^12^ but it is not currently used widely in the UK and there have been no clinical trials conducted in the National Health Service (NHS). Therefore, the aim of the KARDS trial was to investigate the clinical and cost effectiveness of KJD compared with KR in a total of 344 UK NHS patients aged 65 or less.^7^

Given that KJD is not widely used across the UK, using this intervention in a clinical trial context was considered potentially challenging in terms of recruitment and delivery, therefore, a process evaluation (PE) was embedded into the design of KARDS.^7^ PE can be used to explore the provision and receipt of interventions as well as aid the interpretation of the results. They can be used to examine the participants views about the intervention; study how an intervention is implemented; help to explain how an intervention could be optimised or why it may have failed; investigate contextual factors that affect an intervention and/or monitor dose to assess the reach of the intervention.^13 14^

Full details of the planned PE including its aims and objectives can be found in the published study protocol.^7^ It was planned that the PE would be conducted using semi-structured interviews in two key phases (1) internal pilot phase and (2) main trial phase. The primary aim of the planned formative PE in the internal pilot phase (phase one) was to maximise participant recruitment to the trial and identify/minimise variation in intervention delivery that could affect outcomes. Specifically, the objectives in phase one were to (1) document individual sites care pathways (for context), (2) engage with key stakeholders (surgeons, recruiting staff, admin staff and patients) to understand their experiences of recruitment and (3) engage with surgical teams to explore their perceived facilitators or barriers to delivering the surgical interventions as per the protocol.

In phase two, it was planned that further participant/non-participant interviews would be undertaken in the main phase of the trial.^7^ However, as outlined below, recruitment to the trial was closed early due to the impact to NHS surgical services post-COVID pandemic, impacting on the planned work.

### Overview of the KARDS RCT

Five NHS sites were opened to recruitment between March 2021 and May 2022. However, only 24 participants were recruited and randomised from one site: KJD (n=11) and KR (n=13). Two participants crossed over treatment allocations that is, one randomised to KJD actually had a KR and vice versa.

The trial closed early due to slow site set-up and recruitment. Both were initially impacted by the COVID-19 pandemic but neither improved once the initial pressures/issues caused by the pandemic had eased due to the long-term impact to NHS surgical and radiological services. The clinical question was still considered to be relevant, but it was evident that KARDS was not feasible to deliver in the NHS climate, therefore the decision was taken to close the trial to recruitment in October 2022. The final KARDS participant was randomised in August 2022, with the final operation taking place in January 2023. Randomised participants were followed-up until the last individual to have their surgery had completed their 3 month postoperative follow-up assessment. Follow-up was completed in April 2023.

The interviews that we completed were part of the planned KARDS PE. The intention and aims of these interviews did not change from the original plan but given that the trial was stopped early, we were unable to complete a full PE. Therefore, although from a smaller sample than was originally planned, we present the findings of the interviews with patients, health professionals and trial staff here.

## Methods

### Design

This descriptive qualitative study used semi-structured interviews to collect data from patients, health professionals and trial staff. The primary focus was to explore their experiences of being involved in the KARDS trial. The study is presented in line with the Standards for Reporting Qualitative Research.^15^

### Participants

#### Staff

All trial staff, including each principal investigator, other participating surgeons, research nurses and any other staff involved in recruitment and intervention delivery at each site were invited by SA (qualitative researcher independent to the main trial team) to take part in two semi-structured interviews. Each member of staff was provided with an information sheet to explain the purpose of the qualitative study and given time to consider their participation before giving consent to do so. They were reassured that their taking part was entirely voluntary and that any information they disclosed would be anonymised before being shared with the trial team, if necessary.

#### Patients

During the consent process for the main trial, all patient participants were also told about the qualitative study and provided with an information sheet. It was explained that, if they agreed, a researcher would contact them to confirm their willingness to be interviewed and arrange a convenient time to discuss their trial experiences, at which point consent to be interviewed was also collected. They were also reassured that this was entirely voluntary and would not affect their clinical care or participation in the trial.

### Data collection

#### Staff interviews

Initial interviews were arranged around the time each site opened to recruitment. A flexible topic guide with prompts (online supplemental information 1) was used to encourage staff to share their expectations, opinions and experiences of the trial as a whole as well as the site’s care pathway and recruitment processes. Second interviews were arranged with staff members who were still available at the end of the planned internal pilot phase at 12 months, and these focused on understanding experiences of recruiting to the trial and delivering the interventions.

Given the COVID-19 restrictions at the time, all interviews were conducted virtually by a member of the team experienced in qualitative interviewing (SA) using Microsoft Teams. Consent for remote interviews was taken verbally and documented on a consent form. Interviews were recorded on Microsoft Teams; video was removed, and audio recordings were transcribed verbatim for analysis. Transcripts were anonymised and checked for accuracy.

#### Participant interviews

Semi-structured interviews using a flexible topic guide with prompts (online supplemental information 2) were used to conduct the participant interviews by telephone. The aim was to explore the participants’ experiences of their involvement in the trial from consent through to operation and (depending on timing of the interview) recovery. Informed consent was confirmed verbally at the start of each interview and documented on a consent form. Interviews were audio digitally recorded on an encrypted recorder and transcribed verbatim for analysis. Transcripts were anonymised and checked for accuracy.

### Analysis

One-to-one semi-structured interviews were conducted by SA, who is a trained and experienced qualitative researcher, independent to the main trial team and based at a different institution.

The planned qualitative approach for the full PE was thematic content analysis underpinned by Normalisation Process Theory to allow for exploration and explanation of the extent to which the interventions were implemented.^16^,^18^ However, given the difficulties with participant recruitment we took a pragmatic approach, using just thematic content analysis based on the work of Braun and Clarke.^19 20^ Both staff and participant interview data were analysed this way to identify patterns or themes using coding of audio-transcript recordings. All transcripts were organised using QSR NVivo V.12. The qualitative researchers (SA and DRE) met regularly to discuss analysis and potential themes before presenting these findings at monthly Project Delivery Meetings and with the Trial Management Group (TMG) so appropriate actions could be discussed and changes implemented as required. This was done because the original plan for the PE was to be formative during the pilot phase of the trial to ensure that processes were working, but as noted the trial ended during this phase so the planned summative PE did not take place. Quotations have been provided to support our interpretation.

### Patient and public involvement

The KARDS patient and public involvement (PPI) group contributed to the overall design of the study^7^ and provided input and feedback on the questions in the interview topic guide.

## Findings

### Participants

#### Staff

Twenty-three members of staff from across the five open sites were willing to take part in an interview. However, due to clinical demands and the ongoing pressures from COVID-19 restrictions, we were only able to complete 11 staff interviews with eight people from two of the KARDs sites (eight initial interviews that is, just after the sites had opened to recruitment and three follow-up interviews at 12 months).

At site one, five initial interviews were undertaken with two Orthopaedic Consultants, one Research Nurse and two Clinical Trials Assistants between May and June 2021 and one in May 2022. The mean duration of these interviews was 42 min (range 29–52 min). Three follow-up interviews took place between May and June 2022. The mean duration of these interviews was 36 min (range 23–54 min). At the second site, three initial interviews were undertaken with one Specialty Doctor, one Research Nurse and one Clinical Trials Assistant between May and June 2022. The mean duration of these interviews was 24 min (range 19–29 min). All participants were given a pseudonym.

It should be noted that at the time of all the staff interviews, although all sites were open, only one was actively recruiting and delivering the interventions to participants.

#### Patient participants

Twenty-three participants agreed to be contacted about the study. We were able to make contact with 19/23 (83%) participants. Eight (42%) either declined or did not respond to the invitation. The remaining 11 (58%) participants agreed to take part in an interview. Eight participants were male and three female. Five participants had a KR and four underwent KJD. One participant was randomised to KR but switched to KJD and one switched the other way. The mean duration of interviews was 25 min (range 16–55 min).

The 11 initial interviews took place between July 2021 and February 2022 approximately 8–12 weeks following either KJD or KR. Follow-up interviews were conducted with four participants from the KJD arm of the trial. These occurred between 3 and 5 months after the initial interview and lasted a mean of 19 min (range 10–24 min). All participants were given a pseudonym.

### Themes

One overarching theme emerged from the data. For both the staff and participants (specifically those who had KJD), their experiences of KARDS were encapsulated by the theme ‘an unexpected journey’. This incorporated many subthemes which capture the roller coaster of emotions, expectations and unexpected challenges that both staff and participants faced over the course of the study. We have also included the experiences of those who had a KR which could be considered as divergent to the overarching theme as they described a much more familiar and linear journey through their surgery and recovery.

First we present the findings from the initial staff interviews undertaken around the time each site opened to recruitment, followed by findings from the staff interviews carried out approximately 12 months later.

### Staff interviews

#### Initial interviews

Three subthemes illustrating the start of the ‘unexpected journey’ emerged from initial staff interview data, collected at the point when sites had recently opened to recruitment:

- an important research question
- potential challenges to recruitment
- participant equipoise

##### An important research question

All of the staff were comfortable with the aim of the trial and felt that the research question was very important. They were able to see the potential benefits of KJD for the younger population and were excited to get underway:

…if it (KJD) works, it will be a game changer to be honest. Overall, I am fascinated to see the outcome of it. (Scott, Research Nurse)

I think it is really important to have other options, particularly if some of the patients are younger… whilst the procedure is a bit more complicated, and it is going to mean that the patient is out of action for longer I think that is going to be quite appealing. (Richard, Clinical Trials Assistant)

…it’s one of those things that you first hear, and you think well this can’t be true… but it is still important to ask, isn’t it? I genuinely think it is a really important question to answer, whatever that answer is, obviously. (Anthony, Orthopaedic Consultant)

##### Potential challenges to recruitment

Given the delays in starting the trial due to COVID-19, the teams felt that there were going to be additional challenges to recruitment. However, they were still optimistic that they would be able to recruit:

…six to twelve months ago I was really very sceptical about it… not about the procedure, but about patient acceptability but I realised that people are happy to try it. (Patrick, Orthopaedic Consultant)

In terms of future recruitment, I am fairly confident… in terms of patients who are eligible and interested I think we could be looking at a few a month*.* (Richard, Clinical Trials Assistant)

##### Participant equipoise

All of the staff described the participant’s responses to being asked to take part and then to randomisation in terms of equipoise and how this highlighted some things they had not expected or had not come across before:

I have realised that there are two groups of patients… those who really have equipoise and they will take on board whatever comes out. And those who had accepted the fact that it would be like the toss of a coin, and they would be happy with what they get and two of them swapped! (Patrick, Orthopaedic Consultant)

Some people just want their pain to go away so they consider the best or easiest option. Some are a little bit more engaged with what the treatments are and how they work… (Richard, Clinical Trials Assistant)

It has amazed me…there are a couple of patients who have just signed up, they are happy for either. (Scott, Research Nurse)

### Follow-up interviews

Three staff members from one site were reinterviewed after approximately 12 months. Their experiences during that time reiterate ‘the unexpected journey’ theme. They felt that they had been on this journey alongside the participants (specifically those who had KJD) and this was captured in two sub-themes: a roller coaster ride and lessons learnt.

#### A roller coaster ride

Staff felt very comfortable and familiar with the pathway that those randomised to KR followed. However, they described a less straightforward route for the participants in the KJD arm of the trial. The rollercoaster of experiences that they described the participants going through including initial excitement and optimism about the KJD procedure followed by concerns about lack of progress and problems with pain also impacted on them as they continued to work on the trial.

These experiences caused some staff to change their perspectives about the trial from positive, to less so, and back again, as they followed participants along their 12 month journey.

…two people both come out as knee replacement, and I felt emotionally relieved… I’d never had that before. It shouldn’t matter to me… but I had an emotional reaction, that these two lovely people weren’t going to go through this discomfort, and I realised then, I was like, ‘Oh, dear, this is, you know, I’m, I’m starting to feel quite negative about the procedure’ (Richard, Clinical Trials Assistant)

I would say that their (KJD participants) recovery is very slow… it’s something that you’ve got to get your head around and when I look back I thought the recovery speed would be far faster. (Scott, Research Nurse)

#### Lessons learnt

Staff used the experiences they were having as a learning opportunity. They admitted that they had not fully appreciated all the potential problems that those having KJD may encounter but this caused them to reflect and revise the way they described the trial to potential participants and the support that participants needed, both while the distraction was in situ and once it was removed.

I stress the fact that the bare minimum time needed for improvement is one year…and you will feel at times that, “Why did I go through this? So, so those are the things that I think have changed. (Patrick, Orthopaedic Consultant)

…focussing on the distractions side of it…when I looked back I thought the recovery speed would be far faster. I feel in the initial stages it may have been our fault where we’ve not informed them of the difficulties in detail but we, we learnt as we went along and we tried to resolve that as best we could. (Scott, Research Nurse)

### Participant interviews

Here we present, firstly, the findings from the initial interviews with patient participants which were undertaken a few weeks postoperatively. Secondly, we present data from the follow-up interviews, which took place approximately three months later.

#### Initial interviews

Five subthemes emerged from the initial participant interview data to illustrate ‘the unexpected journey’ theme:

- It was an easy decision to take part
- A secret preference
- Recovery following total knee replacement
- Life with the fixator on
- Managing expectations

##### It was an easy decision to take part

Initially, all of the participants felt that they were given adequate written and verbal information about the trial to make an informed choice about taking part. Most of the participants discussed the trial with their spouses/partners and some talked about it with their own general practitioner’s, but all felt that the decision to take part was an easy one:

Rather than getting cut open and going through all stuff … obviously there would have been a recovery after the new process, but I were quite sort of willing to go through that, really. (Brendan, KR)

It was easy yeah, because I knew I’d get something sorted, or I’d get on the way to getting something sorted. (Patricia, KJD but had KR).

I made it (my decision) fairly quickly but mainly in consultation with my wife. Obviously, I couldn't speak to anybody else who'd had it done, 'cause it was new anyway. (Karl, KJD)

##### A secret preference

The participants were able to explain the randomisation process and were very aware that they could receive either intervention. Some reported that they really did not mind which intervention they were randomised to and were just glad to be involved and getting some treatment for their knee. However, a few did admit to having a secret preference and on the whole that was a preference for KJD although there were some exceptions to this:

You know you’re quite happy to go ahead with either procedure… but my preference, yeah love to have my own cartilage back… (Damien, KJD)

I was hoping that I’d get the distraction, yeah. (Brendan, KR)

I felt a bit more uncomfortable with the knee distraction. I don’t know why. I just did. Anyway, it ended up… it came out that it were a knee replacement…it were a relief for me a little bit, yeah. (Sarah, KR)

##### Life with the fixator on

Some participants having KJD reported being shown pictures of the “Meccano” or fixator prior to surgery but for most it was still a shock to see it on their own leg post-operatively. They thought they had been given enough information about KJD when they consented to take part, but with hindsight, there were a lot of things they had not considered or thought about. It was felt that more information about the practicalities of living with a frame would have been very helpful including things such as getting in and out of a car, toileting and having to sleep in one position (on their back) for 6 weeks:

Sleeping was a horrendous experience because I could only sleep on my back… so I was snoring, so my partner ended up going in the spare room. (Damien, KJD)

I didn’t really go out the house apart from into the garden… towards the end of the six weeks I had a couple of car journeys where I shuffled along the backseat… and that was the only way I felt comfortable. I wasn’t given any advice on that type of thing; it was all trial and error. (Karl, KJD)

The participants also described issues with pain, not having enough dressings, problems with clips, and pin site infections:

The pain changes throughout the whole period. It didn’t help that this frame should have had red clips to hold the dressings in place. And I had a mixture of red and blue… but the blue ones would just fall off. And all your dressings fell off. (Brian, KJD)

But I know mine were bleeding from the pins sometimes. I were unlucky enough to get an infection in the pin site. (Phil, KJD)

##### Recovery following knee replacement

Given that KJD is a new intervention, a lot of the focus of the planned PE was directed towards it, but it was also important to capture data from those who had KR to understand their experiences of their procedures as well.

Those allocated to KR were interviewed approximately 6 weeks after surgery. Contrary to the ‘unexpected journey’ theme, the majority reported their recovery to be as they expected and were pleased with their improvements in pain and mobility and the progress they had made:

It’s brilliant. There’s no pain at all. Getting downstairs, I used to take steps one at a time but generally I can go up and down steps quite normally. (Brendan, KR)

I've got really good movement. And, and I've actually gone back to Pilates. I can now get down on the floor. (Carol, KR)

I can do more than I can't. I don’t fall over. I can sleep through night. I've no pain. As far as my job goes, I'm back at work on Monday which is a bit earlier than they expected. (Chris, KR)

##### Managing expectations

While the recovery journey for those who had a KR was relatively straight forward, it was very different for those who had KJD. Participants were interviewed within a few weeks of having their frames removed. They reported that their expectations for their recovery did not match what was happening in reality. Whereas they expected to be “back to normal” (Phil, KJD) or “up and walking within a week” (Damien, KJD) the reality was that their knee was extremely stiff, swollen and painful which led to frustration and worry:

I just want to be out and exercising again because it’s frustrating now, ‘cause I’ve been stuck in like for over ten weeks, really. (Phil, KJD)

It’s six weeks, it’s maybe ‘cause I’m feeling a bit down … maybe in three weeks’ time it’ll all be behind me. When it got to last Thursday it was coming off and in my mind I was free. But I can’t bend my knee. (Dave, KJD)

…it leaves you feeling fairly stressed, because as I’m trying to get mobility back and bend it, and I am in pain, and occasionally in, you know… could I actually be destroying what’s been done? And the fear of that is, yeah, it’s not very good. (Brian, KJD)

Rehabilitation after removal of the fixator was another cause of concern and frustration as in the majority of cases, it did not meet the participant’s expectations:

I eventually got a face to face physio appointment cause I had two over the phone. It has been very frustrating. (Damien, KJD)

I thought there may have been more, more hands-on physio…I've only been in once in the last 4 weeks … and they didn’t, even put me through anything, they just sort of checked it but they didn’t sort of put me through a regime while I was there. (Karl, KJD)

### Participant interviews

#### Follow-up interviews

Four of the participants who had KJD took part in a follow-up interview approximately three months after their initial interview. Two felt that things had improved although slower than they expected, but the other two continued to express frustration and disappointment with their progress in their ‘unexpected journey’. This was encompassed in the sub-theme: a slow recovery.

##### A slow recovery

Very frustrated. It’s just taking so long. I’m still on two crutches. (Damien, KJD)

Walking’s the main hobby which is, it’s pretty pain free now. And I also do quite a bit of crown green bowling. I'm probably in a better place than I was before I had it done. (Karl, KJD)

I didn't think and I don’t think the team did either that it would take just quite as long to get your mobility back properly. (Dave, KJD)

## Discussion

This qualitative interview study was part of the planned PE that was embedded in the first RCT of KJD versus knee replacement in the UK. The aim was to identify potential barriers to recruitment, and any challenges experienced in maintaining the integrity and fidelity of the interventions.^7^

Given the delays in starting the trial due to COVID-19 and due to the trial being stopped early, the PE data were not completed and therefore the findings we have presented are limited as we were only able to interview staff from two sites, of which only one had recruited and randomised participants. However, the information from this, the first qualitative study involving patients undergoing KJD, gave us a lot of insights into patients and staff member’s perceptions, experiences and expectations of the KARDS trial and the ‘unexpected journey’ that a lot of them went on.

On the whole, this small sample of adults under 65 years with knee OA were very open to the idea of having KJD as an alternative to KR and were willing to participate in the trial to be randomised to either procedure. Their reasons for wanting to participate were similar to those in Najafi *et al*’s^21^ qualitative study exploring the factors affecting decision-making for knee arthroplasty: the wish to return to normal life; living without pain; encouragement and support from others including family and friends; and trust in surgical advances. This interest in an alternative to KR was evident among the KARDS participants and also our PPI group, where it was commonly reported that the potential to keep their own cartilage or retaining their own knee was very appealing.

However, implementing and delivering new surgical techniques or treatment innovations either clinically or as part of a clinical trial can be very challenging and often rely on an interplay of factors (structural, organisational, patient-level, provider-level and innovation-based).^22^ This was true for the KJD arm of the KARDS study and early on from the participant interviews it was clear, and was a key learning, that the type of information they were given was not sufficient to manage their expectations about living with an external fixator for six weeks and their recovery following KJD.

Although limited in number, previous trials of KJD versus total knee arthroplasty,^10 23^ and prospective evaluations of KJD^11 12^ did not include or report any PE or qualitative findings exploring the participant experience of the procedure. However, similar to some of the experiences of the KARDS participants, they did report adverse events or complications primarily related to pin site infections but also reduced knee flexion following removal of the fixator which was found to fully normalise after 6–12 months.^11 12 23^ This information about possible time to achieve range of movement and, therefore, return to ‘normal’ activity is crucial to patients undergoing this procedure. This is the type of information that the early KARDS participants felt, with hindsight, was missing when they were informed about the trial and may have helped manage their expectations more successfully during the early phase of their recovery. This information was fed back to the TMG for discussion and consideration, as planned in the formative phase of the PE, but due to the early trial closure, information for participants was not updated.

The majority of those who had KR in the KARDS study experienced a straightforward journey through their treatment and recovery. However, the ‘unexpected journey’ for those who had KJD is reflected in and comparable to themes from Burger *et al*’s^24^ interpretative phenomenological analysis of older people’s lived experience of a hip or knee replacement within a fast track programme. In particular, ‘Finding ways to cope’ and ‘Transition between independence and dependence and back’ echo the KARDS KJD participants’ experiences of the journey they went through and were still going through at the time of their interviews.

Similar to work by Modin *et al*^25^ exploring the impact of Ilizarov external fixation following distal tibia fracture, the KARDS patient participants told us about the imaginative and creative ways they found to cope while living with the fixator on and how this took them from a state of independence to dependence on others and then slowly back again once the frame was removed and they began to recover. All of the hints and tips and real experiences that the participants shared about living with the fixator including how to deal with dressings and pin sites, sleeping positions, getting in and out of a car and using the toilet are crucial to share with potential participants in any future work of this kind. Their struggle with recovery also alludes to the importance of and need for an embedded rehabilitation programme as part of any future clinical trials.

## Conclusion

We were unable to complete the full planned KARDS PE, but where possible, providing adequate and comprehensive information about all aspects of treatment including estimated timelines of recovery are essential in clinical trials of novel interventions. Incorporating a comprehensive rehabilitation package following KJD was also a key learning. PEs in these trials are essential to determine these types of issues as early as possible so appropriate changes can be made to ensure participants have a smooth journey through the trial experience.

## supplementary material

10.1136/bmjopen-2023-083069 online supplemental file 1

10.1136/bmjopen-2023-083069 online supplemental file 2

## Data Availability

Data are available upon reasonable request.
