# Supplementary material for: ‘The unexpected journey’: a qualitative interview study exploring patient and health professionals experiences of participating in the knee arthroplasty versus joint distraction study (KARDS)
Source: BMJ Open. 2024 Jul 11;14(7):e083069. doi: 10.1136/bmjopen-2023-083069 (PMC11253130; doi:10.1136/bmjopen-2023-083069)
Supplement: online supplemental file 1 [file bmjopen-14-7-s001.pdf]

SUPPLEMENTARY INFORMATION 1

Staff Interview Topic Guide

Introduction

- Researcher to introduce themselves and give a brief overview/recap of the aims of the qualitative study.
- Explain what will happen during the interview
- Discuss confidentiality and explain that the interview will be audio recorded.
- Explain that the interviewer was not involved in setting up or designing the trial.
- Ask if the interviewee has any questions and then obtain informed consent.

Professional experience and role on KARDS

- Can you tell me a bit about yourself from the point of view of your professional background/role at the hospital?
- And what is/will be your role on the KARDS trial?

Expectations of KARDS and experience of set-up

This section should only be covered in early interviews (during or shortly after set-up), or in later interviews if the interviewee has only recently become involved in the KARDS.

- When did you first hear about KARDS?
- How do you feel about the research question? Is it important?
- What do you think are the best aspects / benefits of KARDS? Explore what made them want to participate, if relevant.
- Do you have any reservations about KARDS? Explore clinical/academic reservations and/or practical concerns.
- How confident are you about recruiting to the KARDS trial? Explore previous experience of trials.
- How do you feel about starting recruitment? [could prompt using ‘prepared’] Explore experience of training and support provided during set-up.
- How do you feel about starting to deliver the intervention? [as above]

- Explore experience of surgical materials and training (if relevant). Remind the participant that their views will be anonymised and the researcher was not involved in designing the training/trial.

Experience of recruiting to KARDS

This section should only be covered in later interviews when the site has been open for some time.

Experiences of recruitment

- How do you think recruitment is going so far? Explore their experience of research and recruitment to the trial.
- Have there been any patients who met the inclusion criteria but you felt were not appropriate for the trial? Explore why they felt this.
- From your perspective, are there any particular facilitators to getting staff and / or patients engaged in the study?
- And are there any barriers to getting staff and / or patients engaged?
- Who takes the lead in explaining the study to patients and how do you (they) go about it?
- Do you have any ideas for how we could support recruitment?

Experience of delivering knee joint distraction surgery

This section should only be covered in later interviews when the site has been open long enough to have treated some participants.

- Could you talk me through what happens when a patient randomised to knee joint distraction has their surgery? e.g. same surgeon performing both procedures, which frames are being used, do both procedures happen in the same theatres?
- Were there any issues in setting up the knee joint distraction?
- Have there been any issues with delivering knee joint distraction?
- Have any changes been made to the way knee joint distraction is delivered since starting the study? Why?
- How do you think patients are experiencing the knee joint distraction?

Overall experience of KARDS

- What has helped you perform your role in KARDS so far?
- What has made it more difficult?
- How does/will the KARDS trial fit with your normal working methods for this patient group?
- How does/will the KARDS trial fit with established clinical pathways?
- What impact does/will recruiting and treating patients for the trial have on the day to day running of the clinic/theatre?

Closing and thanks

- Is there anything else you'd like to add about your experiences of the study?
- Conclude the discussion and thank the participant for their time and contribution.
