# Supplementary material for: ‘The unexpected journey’: a qualitative interview study exploring patient and health professionals experiences of participating in the knee arthroplasty versus joint distraction study (KARDS)
Source: BMJ Open. 2024 Jul 11;14(7):e083069. doi: 10.1136/bmjopen-2023-083069 (PMC11253130; doi:10.1136/bmjopen-2023-083069)
Supplement: online supplemental file 2 [file bmjopen-14-7-s002.pdf]

SUPPLEMENTARY INFORMATION 2

Participant Interview Topic Guide

Welcome and Introduction

- Ask if the participant has any questions.
- Obtain informed consent and document on the Telephone Interview Consent Form.
- Briefly re-cap on the aims and purpose of the interview.
- Explain what will happen during the interview and how long it is likely to take.
- Emphasise that there are no right and wrong answers, we are interested in understanding their experience.
- Explain that the interviewer has no involvement with the trial itself or their clinical care.

Recruitment questions – participants and decliners

NOTE: This section can be omitted if the participants has already been asked these questions in an earlier interview.

- I'd like to ask you about being invited to take part in the KARDS study. Can you talk me through how you were first informed about the trial?
    - Who was there?
    - What else was going on in the appointment?
    - Can you remember what they said?
    - Can you remember what your first reaction was?
- How did you decide whether to take part in the study?
    - How did you make your decision?
    - What things were important to you?
    - Did you speak to other people about the decision? If so, who?
    - When did you make the decision?
    - Did you find it an easy decision to make? Why/why not?
    - Decliners: what were your reasons for not taking part? Were there any other reasons? How did you feel about saying no? (Reassure the participant that they can speak freely and that there is no pressure on

them to change their decision. Bear in mind that they may not be able to articulate why exactly they didn't want to take part).
    - Participants: what were your reasons for deciding to take part? Were there any other reasons?
- Do you remember being given some written information about the study?
    - What did you think about that information?
    - Was it helpful?
    - How did you use it?
    - Do you have any suggestions for how it could be improved? If so, how?
- Would you have liked information about anything else?
    - If so, what?
    - Why was that important to you?
- Can you tell me what you understand the study to be about?

Trial experience questions – participants only

- I'd like to hear now about your experience of having knee replacement/knee joint distraction as part of the KARDS trial
    - Which treatment did you have?
    - Did you have a preference for either of the treatments before you were randomised? If so why?
    - How did you feel when you found out which treatment you would be getting?
    - Was the treatment like you expected it to be?
    - Has anything been better / worse / different to your expectations?
    - What advice would you give to someone considering this treatment in future?

Interview closure

- Is there anything that we haven't covered in the interview that you think we should know or think about?

Conclude the discussion and thank the participant for their time and contribution.

If the interview is being done shortly after discharge ask if they would be happy to be re-contacted at 3 months.
