# Supplementary material for: ‘The unexpected journey’: a qualitative interview study exploring patient and health professionals experiences of participating in the knee arthroplasty versus joint distraction study (KARDS)
Source: BMJ Open. 2024 Jul 11;14(7):e083069. doi: 10.1136/bmjopen-2023-083069 (PMC11253130; doi:10.1136/bmjopen-2023-083069)
Supplement: Supplementary file 3 [file bmjopen-2023-083069.reviewer_comments.pdf]

PEER REVIEW HISTORY

BMJ Open publishes all reviews undertaken for accepted manuscripts. Reviewers are asked to complete a checklist review form (**http://bmjopen.bmj.com/site/about/resources/checklist.pdf**) and are provided with free text boxes to elaborate on their assessment. These free text comments are reproduced below.

ARTICLE DETAILS

**TITLE (PROVISIONAL)**	'The Unexpected Journey': A qualitative interview study exploring patient and health professionals experiences of participating in the Knee Arthroplasty versus joint Distraction Study (KARDS)
**AUTHORS**	Arnold, Susanne; Pandit, Hemant; Croft, Julie; Stocken, Deborah; Ellard, David; Trial Team, KARDS

VERSION 1 – REVIEW

**REVIEWER**	Lane, Judith<br>Queen Margaret University, School of Health Sciences
**REVIEW RETURNED**	09-Feb-2024

**GENERAL COMMENTS**	Thank you for sharing your work. It is good to see qualitative research coming forward for consideration to help clinicians and researchers develop a better understanding of the healthcare experience. There are some important themes that arise from this study which could be relevant to clinicians or other researchers considering KJD<br><br>The introduction clearly sets the context for the KARDS study. There are some general comments about process evaluation (p. 4/5) however I would like to see some clearer statement of the specific aims of this qualitative element of the study. There is reference to the KARDS protocol however this published protocol is focused on the quantitative aspect. Clearer identification of what the researchers hoped to achieve through the qualitative aspects of the study would help the reader to better understand the methods and results. This is especially needed as the original intention of the process evaluation was to inform the second phase but as the study ended in the pilot phase, the intention of the qualitative aspect may have changed.<br><br>According to the SRQR Standards, there is a need to identify the qualitative approach and research paradigm. This is could be more specifically identified. P.7 simply states that the design is a 'descriptive qualitative study'. Further detail would help to also justify the thematic content analysis approach.<br><br><br>Some further information about who carried out the study recruitment is required, particularly for staff. The reader needs to understand the relationship between staff participants and researcher(s) and be reassured that there was no potential for coercion. Were staff participants made aware that participation was entirely voluntary? Additionally, identification of any other key ethical issues related to the qualitative study needed to be stated particularly in relation to any sensitive information. Staff were asked about their expectations and and concerns about the study - how

	were they reassured that they could be open and honest about their experiences (trustworthiness)?<br><br>The SRQR also asks that research characteristics and reflexivity are outlined. Further information on SA and DE who are identified as the key authors involved in data collection and analysis would be appropriate.<br><br>The results use quotes which are informative and illustrate the themes well. Some quotes are some what repetitive and could be reduced to help with clarity. The start of the section could better signpost the reader to the structure of the results. Would the authors consider if there is a need to separately report initial and follow up interviews? simplifying the results would make it easier for the reader.<br><br>The discussion makes reference to the 'hints and tips' of living with a fixator. Is there no previous literature of patient experiences of living with an external fixator for other lower limb conditions? Linking with similar literature or indicating the lack of literature in this area would also help to inform future research. Summarising the hints and tips in the results could be useful.<br><br>Item 15 on the SRQR asks about techniques to enhance credibility and trustworthiness of the data. This needs to be further addressed. The checklist suggests that this is included on p.7/8 but it is hard to see where it is included.

**REVIEWER**	Hutchinson, Anastasia<br>Deakin University
**REVIEW RETURNED**	29-Feb-2024

**GENERAL COMMENTS**	This is an interesting paper that highlights the importance of conducting process evaluation alongside RCTs. Although the sample size is small and due to early closure of the study only one study site contributed participants, the findings highlight important issues related to: informed consent and provision of comprehensive information about possible extended recovery journeys, and the priorities of patients (if fully informed some participants may well have decided to have the TKR surgery, as they may have preferred a shorter recovery time).<br>Although the protocol has been published previously, more background detail explaining the differences between the two surgical procedures and anticipated recovery pathways would have strength both the abstract and the introduction to this paper. Diagrams illustrating the difference between the two procedures would be useful in the Methods. A stronger summary of prior evidence and the need for an RCT would also make a more compelling argument for the importance of this study.<br>The methods and analysis of the study findings are comprehensively and clearly presented. Some commentary of whether Joint Distraction Surgery is now considered a viable option for younger adults with knee osteoarthritis and whether there is still a need for future RCT would strengthen the Discussion.

VERSION 1 – AUTHOR RESPONSE

Reviewer: 1

Dr Judith Lane, Queen Margaret University

Comments to the Author:

Thank you for sharing your work. It is good to see qualitative research coming forward for consideration to help clinicians and researchers develop a better understanding of the healthcare experience. There are some important themes that arise from this study which could be relevant to clinicians or other researchers considering KJD.

Thank you very much for these comments.

The introduction clearly sets the context for the KARDS study. There are some general comments about process evaluation (p. 4/5) however I would like to see some clearer statement of the specific aims of this qualitative element of the study. There is reference to the KARDS protocol however this published protocol is focused on the quantitative aspect. Clearer identification of what the researchers hoped to achieve through the qualitative aspects of the study would help the reader to better understand the methods and results. This is especially needed as the original intention of the process evaluation was to inform the second phase but as the study ended in the pilot phase, the intention of the qualitative aspect may have changed.

Thank you. We have added some extra detail about the aims of this qualitative work (Page 5) to the manuscript. We have also made it clear that whilst we couldn't complete the full process evaluation as planned, this work represents the results of what we were able to achieve with the patients that were available and this was all part of the formative part of the planned process evaluation. Therefore, the intention and aims of this work did not change from the original plan for the process evaluation but we can only report a shorter qualitative piece rather than a full process evaluation. As we engaged with this smaller than planned group of staff and patients we felt that it was still very important that we reported these findings in this way (Page 6).

According to the SRQR Standards, there is a need to identify the qualitative approach and research paradigm. This is could be more specifically identified. P.7 simply states that the design is a 'descriptive qualitative study'. Further detail would help to also justify the thematic content analysis approach.

Thank you. We have added some more detail to the paper about what the planned approach was i.e. Thematic Content Analysis underpinned by Normalisation Process Theory (Page 8). But again we have highlighted that due to the number of participants involved, we had to take a pragmatic approach to how we analysed and reported the available data (which was not a full process evaluation) and therefore, used a thematic content analysis approach.

Some further information about who carried out the study recruitment is required, particularly for staff. The reader needs to understand the relationship between staff participants and researcher(s) and be reassured that there was no potential for coercion. Were staff participants made aware that participation was entirely voluntary? Additionally, identification of any other key ethical issues related to the qualitative study needed to be stated particularly in relation to any sensitive information. Staff were asked about their expectations and concerns about the study - how were they reassured that they could be open and honest about their experiences (trustworthiness)? The SRQR also asks that research characteristics and reflexivity are outlined. Further information on SA and DRE who are

identified as the key authors involved in data collection and analysis would be appropriate.

Thank you for this comment – we have added more detail about how staff were approached to participate in the study and how we ensured trustworthiness i.e. the researchers (SA and DRE) were independent of the main trial team and work at a different institution and any feedback to the main trial was anonymised (Page 7).

The results use quotes which are informative and illustrate the themes well. Some quotes are somewhat repetitive and could be reduced to help with clarity. The start of the section could better signpost the reader to the structure of the results. Would the authors consider if there is a need to separately report initial and follow up interviews? simplifying the results would make it easier for the reader.

Thank you for this comment. As suggested, we have reduced some of the quotes (Pages 11-19) and we have added more information to the start of the sections (Pages 11 & 14) to guide the reader as to the structure of the sections and changed some of the sub-headings to make it easier for the reader to follow (Pages 11, 13,14,15,18).

The discussion makes reference to the 'hints and tips' of living with a fixator. Is there no previous literature of patient experiences of living with an external fixator for other lower limb conditions? Linking with similar literature or indicating the lack of literature in this area would also help to inform future research. Summarising the hints and tips in the results could be useful.

We struggled to find a lot of qualitative work about patient experiences of living with an external fixator but have added a reference by Modin et al about postoperative impact of daily life after primary treatment of proximal/distal tibia fracture with Ilizarov external fixation (page 21). We have also summarised some of the hints and tips that could be useful (page 21).

Item 15 on the SRQR asks about techniques to enhance credibility and trustworthiness of the data. This needs to be further addressed. The checklist suggests that this is included on p.7/8 but it is hard to see where it is included.

Thank you for this comment – as above we have added more detail about how we ensured trustworthiness i.e. the researchers (SA and DRE) were independent of the main trial team and work at a different institution and any feedback to the main trial was anonymised (Page 7). We have also added more detail about regular meetings between SA and DE to discuss analysis and potential themes prior to feeding back to the TMG and that we have included quotations to support our interpretation of findings (Page 9).

We have updated our SRQR table to reflect the changes that we have made.

Reviewer: 2

Dr Anastasia Hutchinson, Deakin University

Comments to the Author:

This is an interesting paper that highlights the importance of conducting process evaluation alongside RCTs. Although the sample size is small and due to early closure of the study only one study site contributed participants, the findings highlight important issues related to: informed consent and provision of comprehensive information about possible extended recovery journeys, and the priorities of patients (if fully informed some participants may well have decided to have the TKR surgery, as

they may have preferred a shorter recovery time).

Thank you very much for these comments.

Although the protocol has been published previously, more background detail explaining the differences between the two surgical procedures and anticipated recovery pathways would have strength both the abstract and the introduction to this paper. Diagrams illustrating the difference between the two procedures would be useful in the Methods. A stronger summary of prior evidence and the need for an RCT would also make a more compelling argument for the importance of this study.

Thank you. We feel that given that this is not the main trials results paper, we have given a good summary of the problem being addressed by the trial. We have guided the reader to the protocol paper more clearly in the introduction section where both the rationale for the trial and interventions are described the KJD intervention is described (Page 4 and 5). We feel that we have provided adequate description of the intervention here and diagrams of the two procedures are not appropriate in this paper but may be in the main results paper.

The methods and analysis of the study findings are comprehensively and clearly presented.

Thank you very much.

Some commentary of whether Joint Distraction Surgery is now considered a viable option for younger adults with knee osteoarthritis and whether there is still a need for future RCT would strengthen the Discussion.

Based on the available data, results are presented with caution and we cannot draw any conclusions about KJD as a viable option for younger adults with knee osteoarthritis and it would be wrong to do so as the trial was not completed as planned. We are hopeful that further RCTs investigating this research question will be able to provide more definitive results.

VERSION 2 – REVIEW

**REVIEWER**	Lane, Judith<br>Queen Margaret University, School of Health Sciences
**REVIEW RETURNED**	12-Apr-2024

**GENERAL COMMENTS**	Thank you for the revisions which I feel provide greater clarity and robustness to the study reporting.
